# Non-contact heart rate estimation based on singular spectrum component reconstruction using low-rank matrix and autocorrelation

**DOI:** 10.1371/journal.pone.0275544

**Published:** 2022-12-30

**Authors:** Weibo Wang, Zongkai Wei, Jin Yuan, Yu Fang, Yongkang Zheng

**Affiliations:** 1 Electrical Engineering and Electronic Information, Xihua University, Chengdu, China; 2 State Grid Sichuan Electric Power Research Institute, Chengdu, China; Valahia University of Targoviste: Universitatea Valahia din Targoviste, ROMANIA

## Abstract

The remote photoplethysmography (rPPG) based on cameras, a technology for extracting pulse wave from videos, has been proved to be an effective heart rate (HR) monitoring method and has great potential in many fields; such as health monitoring. However, the change of facial color intensity caused by cardiovascular activities is weak. Environmental illumination changes and subjects’ facial movements will produce irregular noise in rPPG signals, resulting in distortion of heart rate pulse signals and affecting the accuracy of heart rate measurement. Given the irregular noises such as motion artifacts and illumination changes in rPPG signals, this paper proposed a new method named LA-SSA. It combines low-rank sparse matrix decomposition and autocorrelation function with singular spectrum analysis (SSA). The low-rank sparse matrix decomposition is employed to globally optimize the components of the rPPG signal obtained by SSA, and some irregular noise is removed. Then, the autocorrelation function is used to optimize the global optimization results locally. The periodic components related to the heartbeat signal are selected, and the denoised rPPG signal is obtained by weighted reconstruction with a singular value ratio. The experiment using UBFC-RPPG and PURE database is performed to assess the performance of the method proposed in this paper. The average absolute error was 1.37 bpm, the 95% confidence interval was −7.56 bpm to 6.45 bpm, and the Pearson correlation coefficient was 98%, superior to most existing video-based heart rate extraction methods. Experimental results show that the proposed method can estimate HR effectively.

## 1 Introduction

Heart rate (HR) is an important indicator to measure human physiological activities, monitoring human health and emotional state. It has been widely used in cardiovascular disease diagnosis, health assessment, and emotional detection [1–5]. The traditional contact HR measurement methods include electrocardiogram (ECG) and photoplethysmography (PPG). Although the measurement accuracy of the two methods is high, the detection type that relies on specific sensors to contact the subjects’ skin is not suitable for patients with skin damage and newborns. In contrast, the non-contact heart rate measurement using microwave Doppler or computer vision technology has attracted more and more research attention due to its non-contact advantages. LU G et al. [6] measured HR using Doppler radar. Pavlidis et al. [7] successfully extracted the HR of the subjects by analyzing the facial thermal infrared images. However, these devices are expensive and require complex hardware support, making it challenging to promote practical applications.

Based on the principle of PPG, remote photoplethysmography (rPPG) can collect the facial skin color changes of subjects by the camera in a non-contact way to extract the PPG signal and detect the HR and its changes related to cardiac activity [8]. Verkruysse et al. first verified the possibility of using facial video to measure HR and found that the color intensity of the facial area collected by ordinary cameras had periodic changes correlated with blood volume pulse (BVP) [9]. Compared with non-contact detection methods such as microwave Doppler and thermal infrared, the cameras reduce the detection cost and operation complexity, which has obvious advantages in unconstrained scenes such as neonatal monitoring [10], fatigue driving judgment [11], and pressure monitoring [12]. However, because the color change of the human face caused by cardiovascular activity is subtle, it will be affected by noise such as illumination change and motion artifact, which makes the measurement accuracy disturbed. Many research methods on rPPG denoising have been proposed in recent years to solve this problem. Blind source separation (BSS) technology is usually used to remove the noise contained in rPPG. Poh et al. decomposed the original RGB three-channel signals into three independent source signals through independent component analysis (ICA) and extracted the HR from the second component [13]. Lewandowska et al. proposed a method based on principal component analysis (PCA) for HR measurement and evaluated the measurement effect under different regions of interest (ROI), different combinations of color channels, and different illumination conditions. The results showed that the accuracy of this method was affected by the environmental illumination variables [14]. Different from the assumption of linear mixing for BSS, based on the two-color reflection model, Haan et al. eliminated the interference caused by the mirror reflection component through the difference between the two orthogonal chromaticity signals. In addition, they also proposed a rPPG method based on the normalized BVP vector signal in the normalized RGB color space to improve the motion robustness [15, 16]. Li et al. proposed an anti-interference method of normalized least mean square (NLMS) adaptive filter to correct illumination change [17]. Chen et al. reduced the influence of environmental light changes by decomposing green channels with the ensemble empirical mode decomposition (EEMD) of the Hilbert-Huang transform [18]. Wang et al. used image redundancy to offset the influence of the facial movement [19]. Kumar et al. solved the problem of low signal-to-noise ratio (SNR) caused by deep skin color and weak illumination conditions by using a weighted average to combine the skin color change signals of different tracking regions of the face [20]. Based on the multi-task convolutional neural network, Yue et al. proposed a framework combining empirical mode decomposition and permutation entropy to reduce the impact of face jitter and shooting environment [21]. Niu et al. designed a transfer learning strategy to estimate HR from the spatiotemporal representation of HR information [22]. In addition, the author further improves the HR estimation method of face videos based on channel and spatio-temporal attention mechanism [23]. Song et al. designed a new pulse wave generation framework based on generative adversarial network to improve waveform quality, thereby improving the accuracy of heart rate detection [24].

This paper proposes a non-contact HR measurement method (LA-SSA) using low-rank sparse matrix decomposition and autocorrelation function for SSA decomposition component selection and reconstruction. The structure of the article is as follows. Section 2 introduces the basic framework of HR detection and elaborates on the basic principle of the LA-SSA method. Section 3 introduces the database information, evaluation index, and the experimental results on the UBFC-RPPG and PURE database. Concluding remarks are given in Section 4.

## 2 Methods

**Fig 1:**
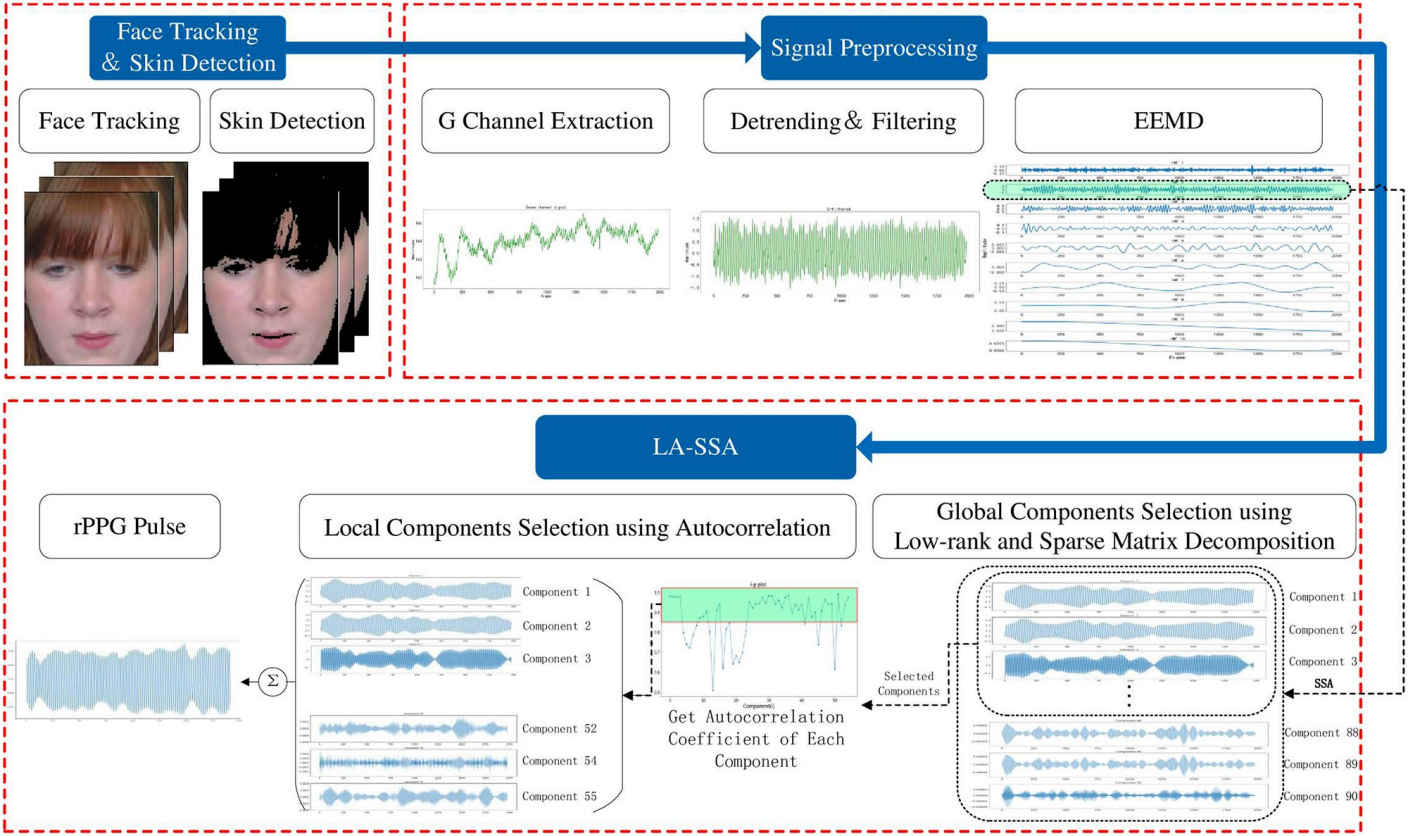
Face video HR estimation framework.

Based on SSA decomposition components, the proposed method introduces low-rank sparse matrix decomposition and autocorrelation function in component reconstruction, which reduces the interference of irregular noise contained in the signal and retains the periodic components of HR correlation. It realizes the accurate extraction of pulse signals containing HR information from facial videos. The framework of this study is shown in Fig 1. Firstly, face tracking and skin detection are performed for each frame of the face video, and the detected facial skin is regarded as the ROI. Then ROI is divided into the three RGB channels. It is known that the HR-related information contained in the G channel is more prosperous than that contained in the other two channels. When the video sample is seriously disturbed by noise, the introduction of the other two channels may bring more noise than the HR information. Therefore, only the G channel is selected in this paper to extract the HR-related information. Spatially averaged over all pixels in the ROI to reduce the noise, and the average pixel value of each frame in the G channel is used to form the original facial signal. The original signal is smoothed and preprocessed by detrending, normalization, and five-point moving average filtering. In preprocessing, EEMD is introduced for rough denoising of the signal. Based on the results of rough denoising, the LA-SSA algorithm is used for further denoising of the signal to extract the HR information.

### 2.1 Face tracking

Since head movement and background noise greatly influence pulse waves, accurate face detection and tracking are the keys to collecting high-quality original pulses. In the literature, the method of Viola-Jones combined with Kanade-Lucas-Tomasi is mainly used to realize the rapid detection of the human face. However, this method has good stability only when the measured object keeps the head still, which is greatly affected by the head movement and prone to false and missed detection. In order to ensure stable face detection in a more realistic environment, this paper uses the multi-task convolutional neural network (MTCNN) [25] model for face detection. The model is based on the idea of candidate box and classification. Through the cascade of P-Net (Proposal Network), R-Net (Refine Network), and O-Net (Output Network), it can realize fast and efficient face and feature point detection.

**Fig 2:**
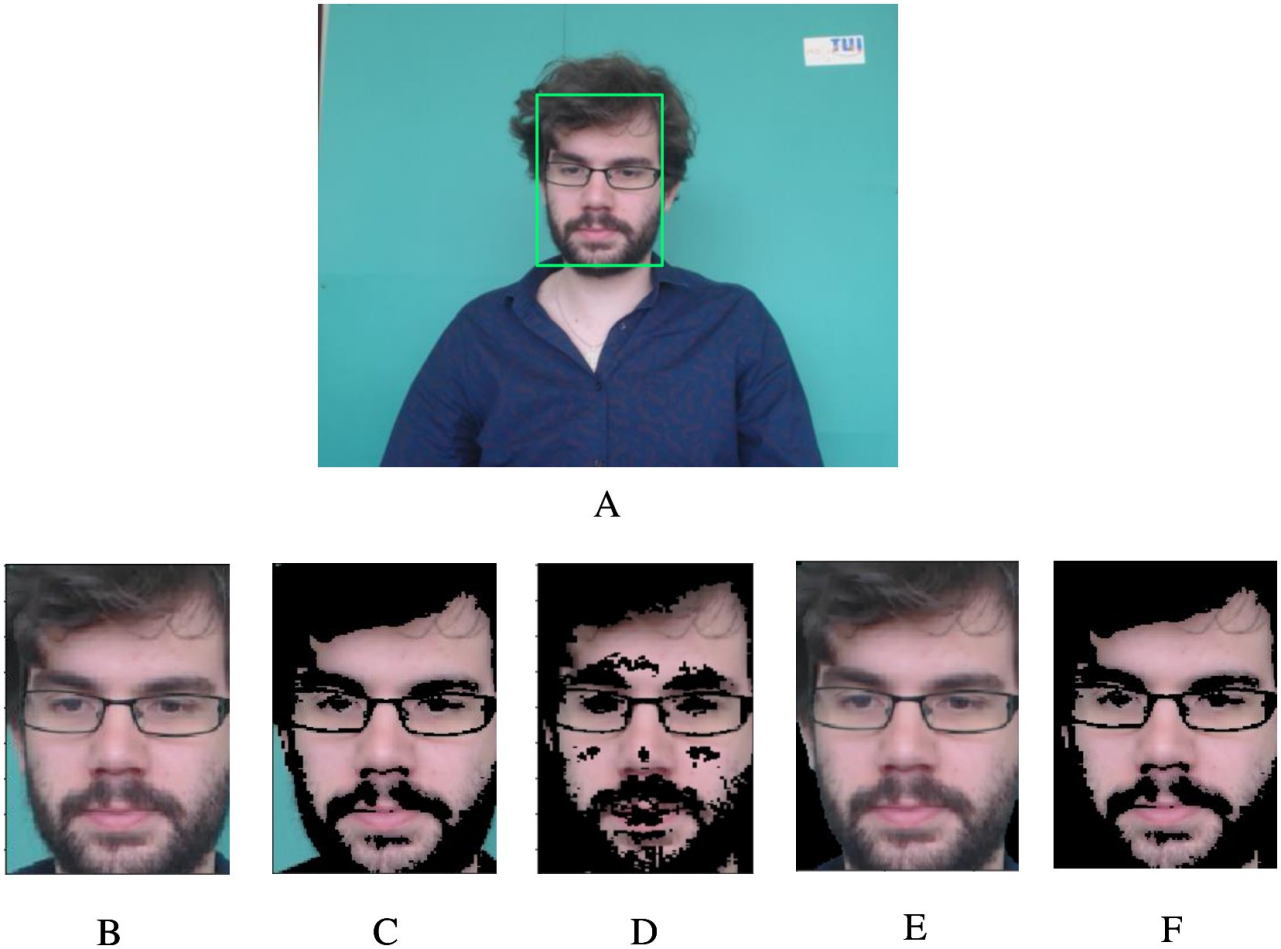
Face and skin detection results. A:The result of face detection. B:The cropped face. C∼F: The skin detection results of RGB, YCrCb, YCrCb+OTSU, RGB-H.

In face detection, the MTCNN face detection model is used to track the face of each frame in the video. If the face region is not detected in the current frame, the face region of adjacent frames is used. However, the results obtained by face detection still contain some non-skin areas, including hair, eyebrows, nostrils, and a small number of background areas, which cannot provide any helpful information related to HR. On the contrary, the eye-blinking and slight movement of lips will introduce artifacts into pulse signals. Therefore, the skin detection algorithm based on RGB-H is applied to exclude non-skin areas as much as possible after obtaining the face area. A frame of the face and skin detection results are shown in Fig 2.

### 2.2 Skin detection based on RGB-H threshold

After the face is detected, ROI is identified in the face to extract information related to cardiac activity. The selection of ROI directly affects the accuracy and reliability of the HR extraction algorithm. Usually, the whole face, the rectangular area of the face cut with a particular proportion, and the forehead, nose, or cheek area can be selected as ROI. Rapczynski et al. proved that the whole facial skin and forehead could obtain more accurate HR information [26]. More skin pixels can improve SNR to obtain a clearer rPPG signal and better HR estimation. Since the forehead may be covered by hair, and the deflection of the head will affect the extraction of ROI, this paper selects the entire facial skin area as ROI to extract HR-related signals. The current skin detection algorithms mainly include threshold-based, model-based, region-based [27], etc. Considering that the skin detection method based on threshold can effectively and accurately detect the skin with less computation and shorter time. By comparing the detection effect of four skin detection algorithms based on the RGB threshold, YCbCr threshold, YCrCb space Cr component + OTSU segmentation, and RGB-H threshold, this paper selects the RGB-H threshold segmentation algorithm to detect the skin of each frame. The whole face skin area detected is used as ROI to realize HR detection.

$$\begin{array}{c} \left\{ \begin{array}{c} R>95\wedge G>40\wedge B>20\wedge \\ \text{max}(R,G,B)-\text{min}(R,G,B)>15\wedge \\ R>G\wedge R>B\wedge \\ 0\leq H\leq 60 \end{array}\right. \end{array}$$ (1)

The H and RGB values of each pixel are compared to the thresholds to judge whether the pixel is a skin pixel. The final skin detection result is obtained by removing non-skin pixels outside the selection criteria.

### 2.3 Preprocessing

In order to minimize the noise component in the signal before LA-SSA decomposition and reconstruction, based on the characteristics of LA-SSA single-channel input, after comparing the HR estimation accuracy of LA-SSA using different inputs such as G channel, signal obtained by CHROM method, ICA filtering results, and EEMD decomposition screening signal, EEMD is introduced into the preprocessing to realize the rough denoising of the signal in this paper. Proposed by Wu et al. [28], the EEMD algorithm is an adaptive time-frequency analysis method. It is based on EMD algorithm and can decompose nonlinear and non-stationary signals into a finite number of intrinsic mode functions (IMFs) according to the time scale characteristics of the signal itself, without setting any basis functions in advance. Meanwhile, by adding white noise to the original signal, this method maps different time scale components to the reference time scale related to white noise, and the white noise is removed by multiple mean, which effectively solves the problem of modal aliasing in EMD. This method has more evident advantages in tracking physiological signals such as pulse period than the existing stationary methods. At present, many studies have used the EEMD algorithm to solve the problem of noise removal in rPPG signals [18, 29, 30].

**Fig 3:**
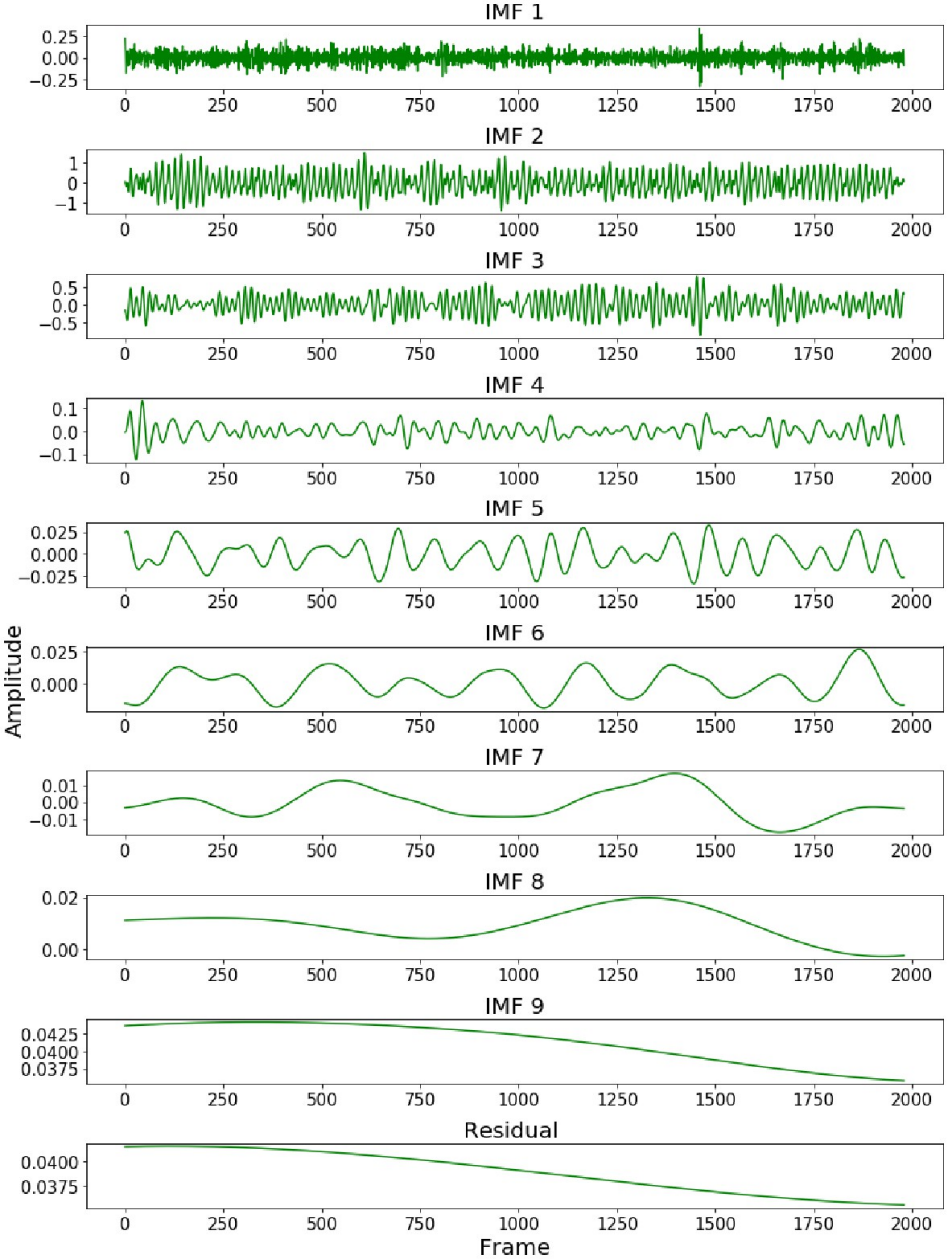
The IMFs and the residual of the filtered signal.

**Fig 4:**
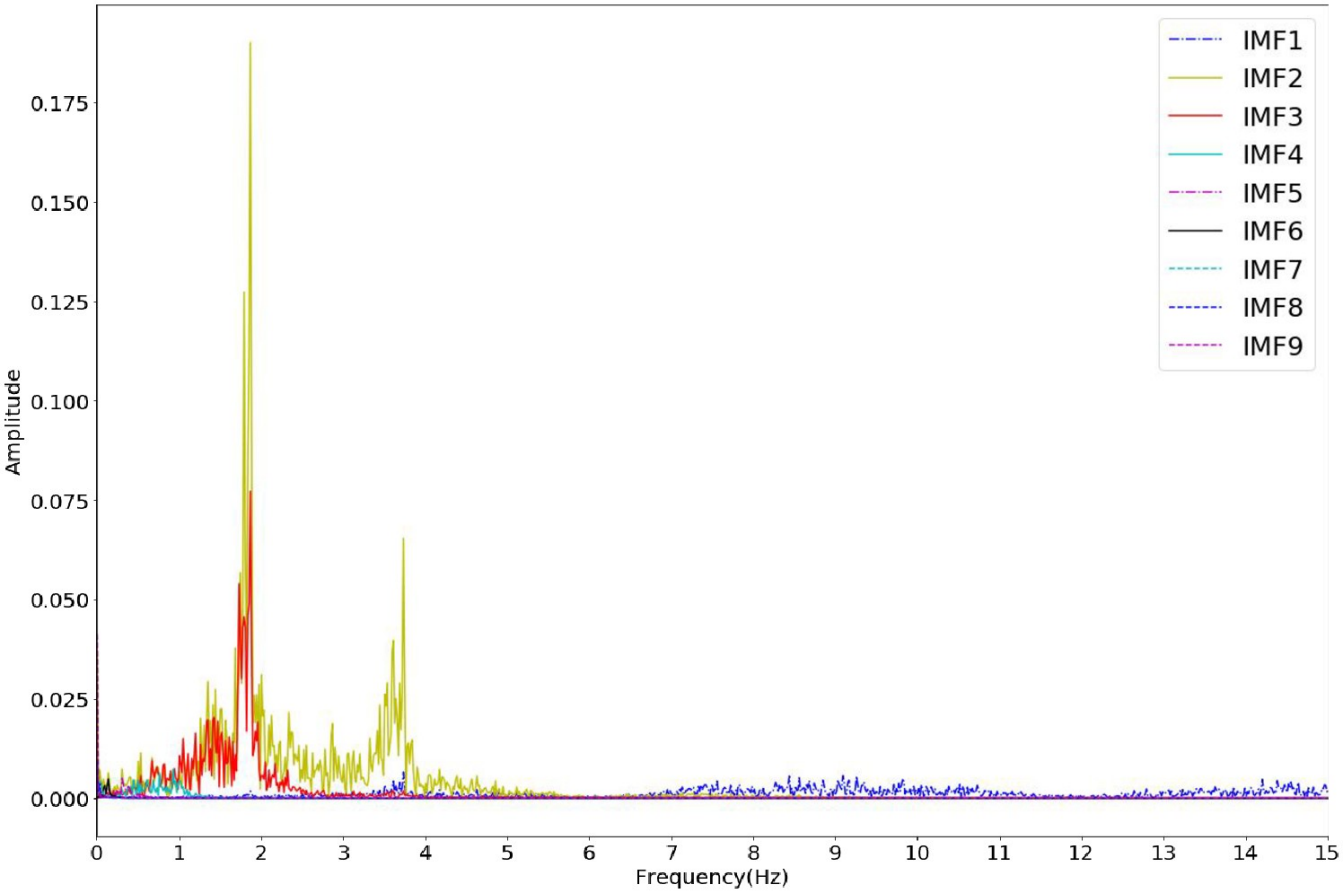
Frequency domain analysis of the decomposed IMF components.

**Fig 5:**
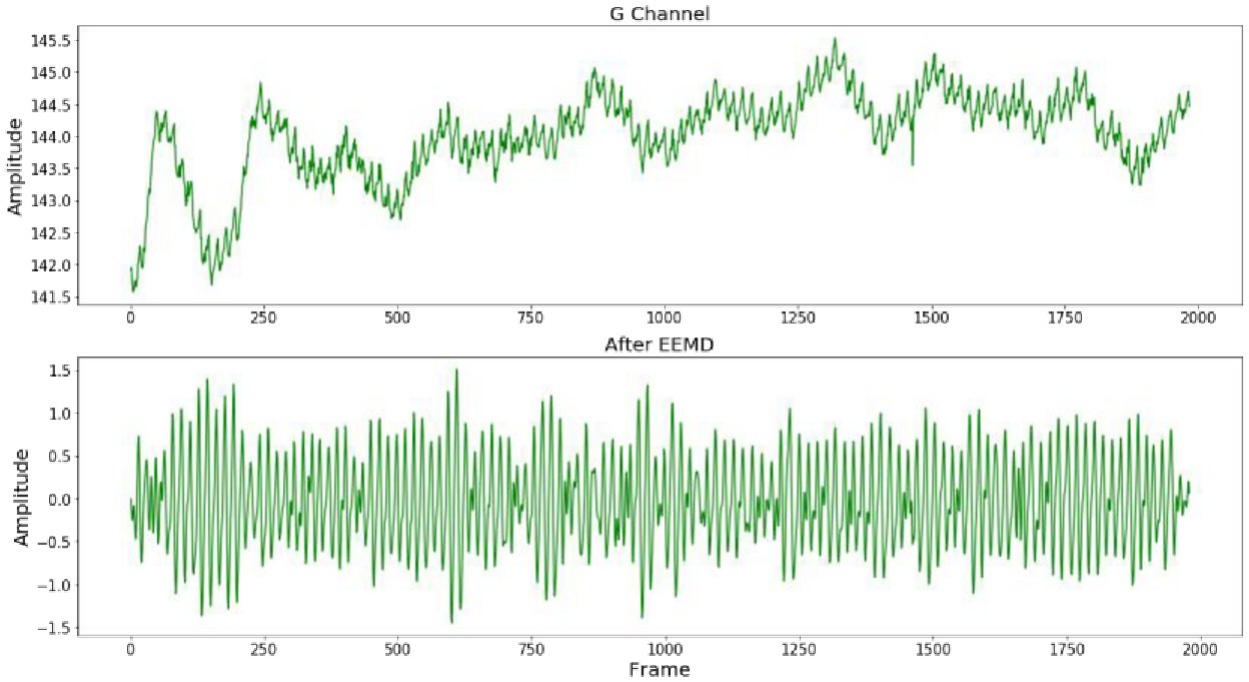
The waveforms of Green channel and after pretreatment.

Firstly, ROI is separated into the three RGB channels. The pixels in the ROI are spatially averaged and the process is repeated for each video frame to form the original facial signal which contains the rPPG signal. The prior smoothing method [31] is used to detrend the original signal to eliminate the low-frequency trend in the signal. The detrended signal is normalized and then smoothed by five-point moving average filtering. The standard deviation of additional noise is set to 0.05, and the average number of ensembles is set to 100. The filtered signal is decomposed by EEMD, and nine IMFs and one residual are obtained, as shown in Fig 3. Since the IMF related to the HR signal cannot be identified in the time domain, the FFT of nine IMFs is carried out. The component that the spectrum contains the highest peak in the heart rate range (0.7 Hz—3 Hz) is selected as the preprocessing output result to realize the rough denoising of the signal. It can be seen from Fig 4. that the target signal is IMF2. Fig 5. shows the extracted G-channel signal and the preprocessed signal obtained after EEMD decomposition and screening. It can be seen that the preprocessed signal is relatively smooth as a whole, and the low-frequency trend is removed, achieving the effect of coarse signal denoising.

### 2.4 Proposed method: LA-SSA

**Fig 6:**
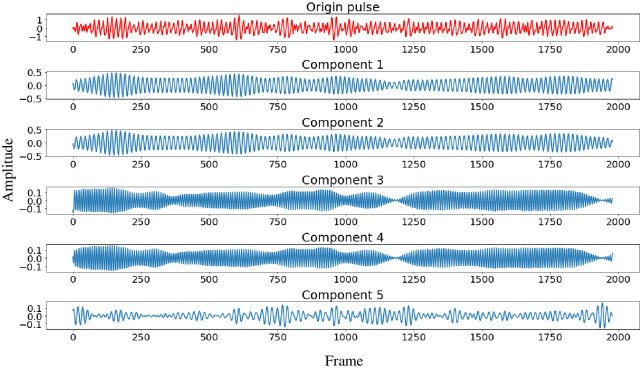
Original signal and its first five components.

**Fig 7:**
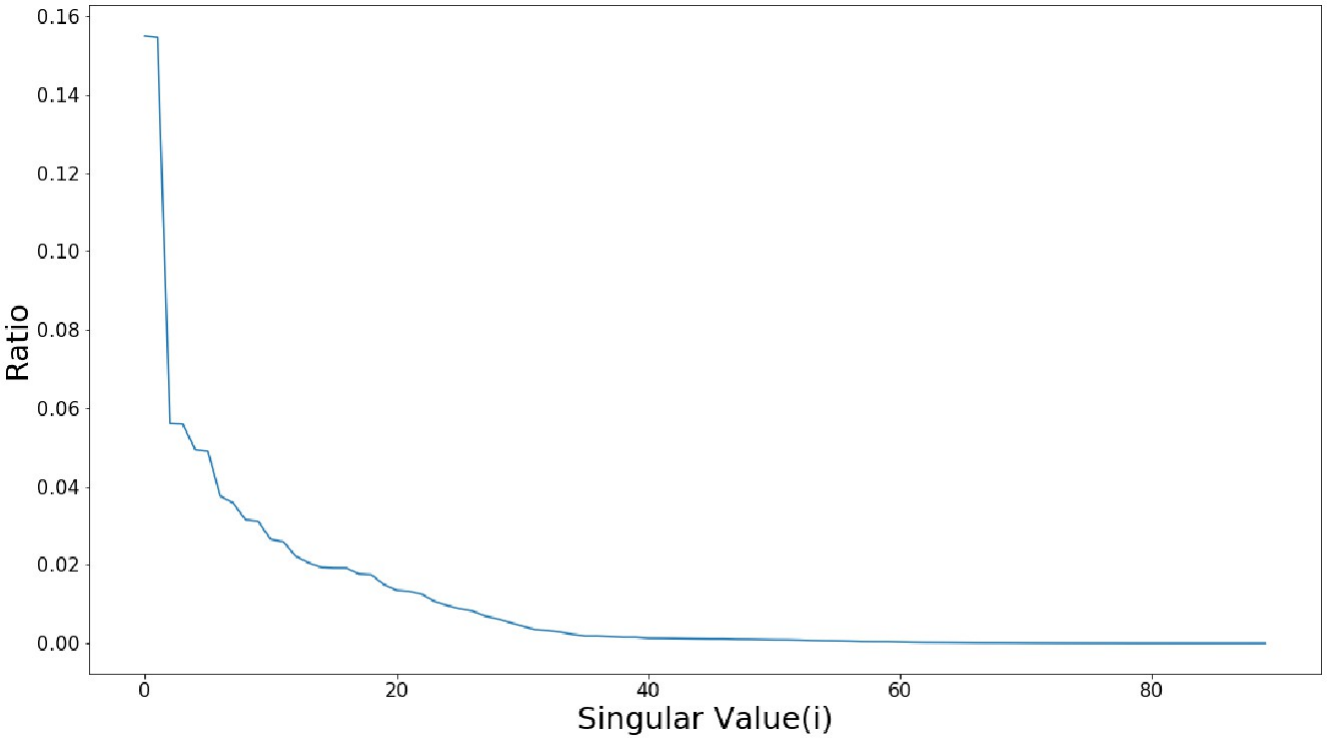
Proportion curves of singular values.

**Fig 8:**
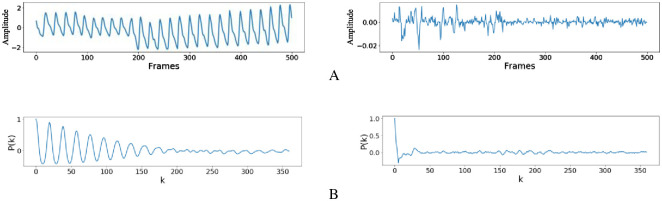
The autocorrelation of HR-related signal and noise signal. A: The HR-related signal(left), the noise signal(right). B: The autocorrelation of HR-related signal(left), The autocorrelation of noise signal(right).

**Fig 9:**
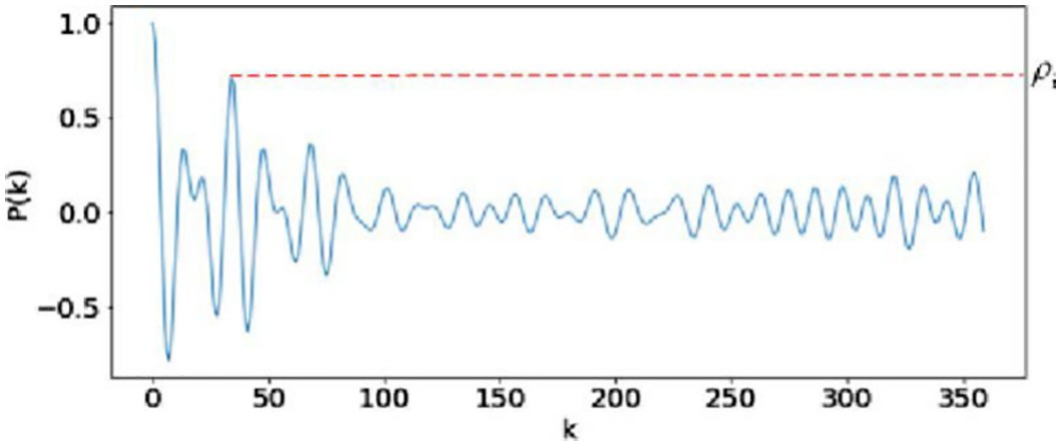
Autocorrelation coefficient *P*_*i*_(*k*) and maximum autocorrelation coefficient *ρ*_*i*_ of component *i*.

**Fig 10:**
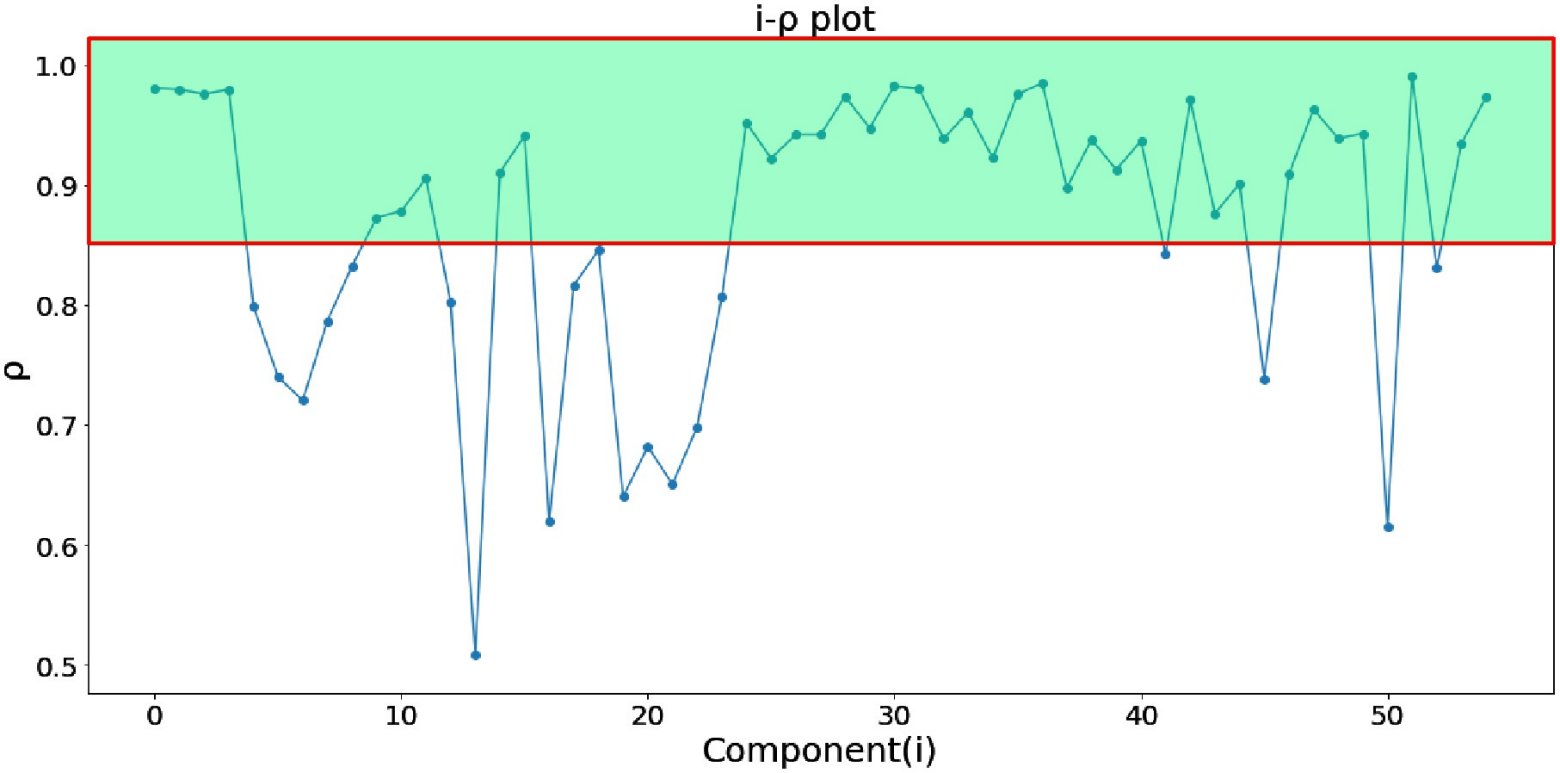
Screening of the first *s* maximum autocorrelation coefficients.

**Fig 11:**
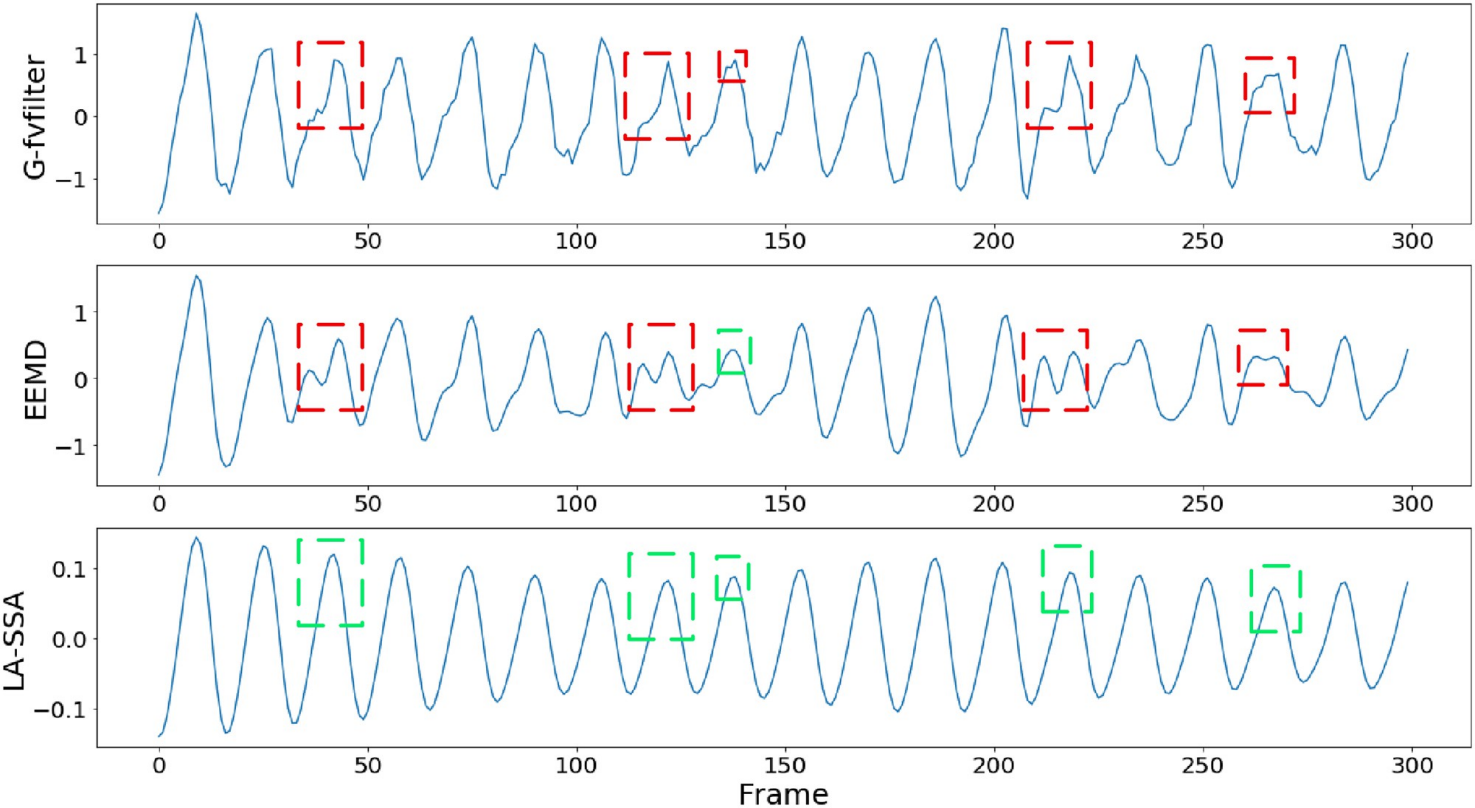
Waveform comparison of filtered G-channel, EEMD and LA-SSA.

**Fig 12:**
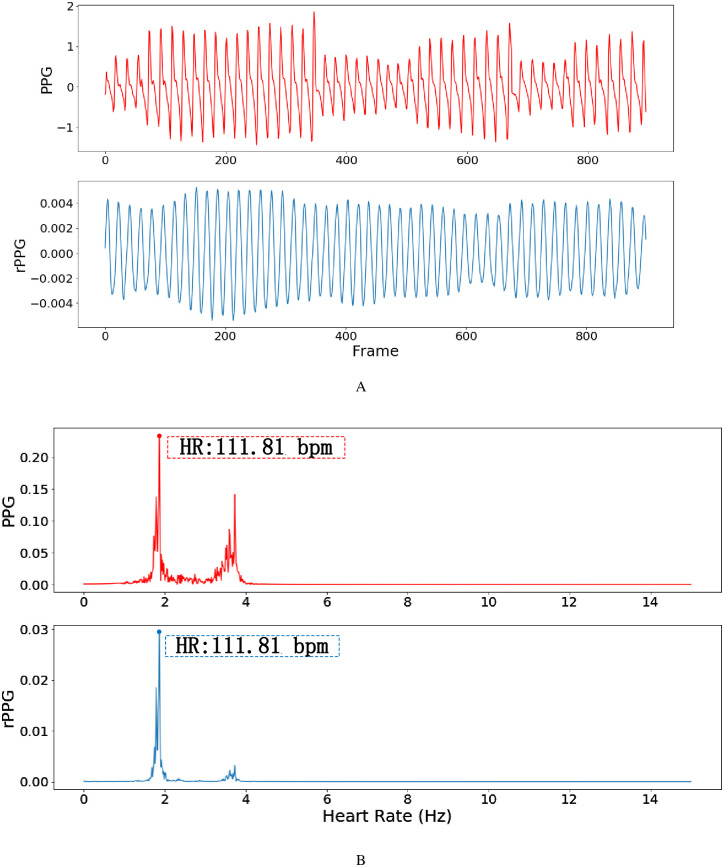
Comparison of LA-SSA result with reference PPG signal. A: Waveform comparison. B: Spectrum comparison.

SSA is a non-parametric decomposition technique, and its calculation is different from the wavelet series expansion or AR algorithm. It neither needs to use the basic function nor assume a specific model. It decomposes the time series into multiple variable components based on the singular value decomposition (SVD) of the time series trajectory matrix, without any prior knowledge of the time series, and achieves the effect of adaptive noise reduction of the signal by grouping, screening and reconstructing the target components [32]. In this section, based on the denoising results of EEMD, the method named LA-SSA is proposed to further denoise the rPPG signal. In the LA-SSA method, the preprocessed signal is decomposed by the SSA method to obtain several related components. Then the signal reconstruction is carried out with less noise by selecting the appropriate components using low-rank sparse matrix decomposition and the autocorrelation function, thereby improving the quality of the rPPG signal. The main steps of the proposed LA-SSA method are as follows:

1. Trajectory matrix construction
  Assuming that the length of time series *x*(*n*) is *N*, the length of the sampling window is set as *L* (1 < *L* < *N*), and the number of windows is *K* = *N* − *L* + 1. The trajectory matrix **X** can be expressed as:
  $$\begin{array}{c} \text{X}=[\begin{array}{cccc} x_{1} & x_{2} & \cdots & x_{K}\\ x_{2} & x_{3} & \cdots & x_{K+1}\\ \vdots & \vdots & \ddots & \vdots \\ x_{L} & x_{L+1} & \cdots & x_{N} \end{array}] \end{array}$$ (2)
  Usually, L is a quarter of the data length [33]. When the data is relatively long and periodic, the best choice for the value of L is the quarter of the longest period in the data. In the rPPG signal, the longest fluctuation cycle caused by cardiovascular-related activities is about 12 s [34]. Therefore, the value of L is set as the number of data points for 3s in this paper.
2. Singular value decomposition
  **X** is decomposed by SVD and expressed as the sum of *d* component matrices. Then **X** can be represented as:
  $$\begin{array}{c} \text{X}=\sum_{i=1}^{d}{\text{X}}_{i}=\sum_{i=1}^{d}\sqrt{\lambda_{i}}u_{i}v_{i}^{T}(i=1,2,\ldots ,d) \end{array}$$ (3)
  Where *d* is the number of non-zero singular values and *d* ≤ min(*L*, *K*), **X**_*i*_ is the i-component matrix of **X**, $\sqrt{\lambda_{i}}$, *μ*_*i*_ and *ν*_*i*_ are the ith singular value of **X** and the corresponding left and right singular vectors, respectively. $\sqrt{\lambda_{1}}\geq \sqrt{\lambda_{2}}\geq \cdots \geq \sqrt{\lambda_{d}}$ is the singular spectrum of trajectory matrix **X**. It is known that the larger the singular value is, the more significant the contribution of the corresponding component matrix to the trajectory matrix is. So operations like eliminating noise by reconfiguring a new signal removing partial component matrix **X**_**i**_ can be performed.
3. Components filtration
  (a) Global optimization based on low-rank sparse matrix decomposition
    In the singular spectrum, it is believed that the component signals obtained by component matrixes corresponding to the first *s* larger singular values contain the primary information of the signal, while the components corresponding to the smaller singular values mainly reflect the noise interference and other components. Fig 6. shows the original signal before decomposition, and the reconstructed signal components corresponding to the first 5 singular values. And the ratio curve of singular values is shown in Fig 7. Obviously, the contribution to reconstructing the original signal increases as the proportion of singular values increases. Therefore, the rPPG waveform can be reconstructed by screening the first *s* components to eliminate noise components unrelated to HR information. It is essential to determine a proper value of *s*, if *s* is too large, part of the noise component will be mixed into the reconstructed rPPG waveform, reducing noise reduction performance, and if *s* is too small, some helpful information related to HR will be eliminated. In this paper, the low-rank sparse matrix decomposition is employed to get the best approximate low-rank matrix **A** of **X**, and the rank of **A** is the value of *s* [35]. Then the global optimization of component matrixes can be realized by selecting component matrixes corresponding to the first *s* singular values. Suppose **X** is affected by random (sparse) noise. In that case, its low-rankness will be destroyed and become full-rank, which makes **X** contains much redundant information besides HR information, thus affecting the accuracy of heart rate detection. then through the low-rank and sparse matrix decomposition, the denoise problem can be described as:
    $$\begin{array}{c} \left\{ \begin{array}{c} \underset{A,E}{\text{min}}\|A{\|}_{*}+\eta \|E{\|}_{0},\eta =1/\sqrt{\text{max}(m,n)}\\ s.t.\text{X}=A+E \end{array}\right. \end{array}$$ (4)
    Where **A** and **E** represent low-rank matrix and sparse noise matrix, respectively, ‖⋅‖_*_ and ‖⋅‖_0_ denote kernel norm and zero norm of the matrix entries, respectively, *m* and *n* represent the number of rows and columns of matrix **X**, respectively. The exact augmented Lagrange multiplier algorithm (EALM) solves this optimization problem (Table 1). The Lagrange function is defined as:
    $$\begin{array}{c} L\left(A,E,Y,\mu \right)=\|A{\|}_{*}+\eta \|E{\|}_{0}+\langle Y,\text{X}-A-E\rangle +\frac{\mu }{2}\|\text{X}-A-E{\|}_{F}^{2} \end{array}$$ (5)
    Where *μ* is the penalty coefficient, 〈**Y**, **X** − **A** − **E**〉 = *Tr*(**Y**^*T*^(**X** − **A** − **E**)), and the initial value of Lagrange multiplier **Y** can be expressed as:
    $$\begin{array}{c} Y_{0}=\text{sgn}\left(\text{X}\right)/\text{max}\left(\|\text{sgn}(\text{X}){\|}_{2},\eta^{-1}\|\text{sgn}(\text{X}){\|}_{\infty }\right) \end{array}$$ (6)
    Where ‖⋅‖_∞_ and ‖⋅‖_2_ represent infinite norm and 2-norm of the matrix entries, respectively. Then the optimal approximate low-rank matrix of **X** can be iteratively solved by the following EALM method:
    Where *T*_*τ*_(*x*) = sgn(*x*) max(|x| − *τ*, 0), *D*_*τ*_(**M**) = **U** *T*_*τ*_(**Σ**)**V**^*T*^. The value of coefficient *ρ* determines the convergence rate, which is usually between 1.1 and 2. It has been proven in [36] that the Lagrange multiplier **Y** is sufficient to guarantee the linear convergence of the EALM algorithm when **X** − **A** − **E** is continuously differentiable. The rank of **A**_**k**_ is the value of *s*, then the global optimization of component matrixes by preserving corresponding first *s* component matrixes of **X** can be performed.
    For the reserved first s component matrixes, each matrix **X**_**i**_ is reduced to the corresponding time series component with length *N* by diagonal averaging (the first 5 corresponding time series are as shown in Fig 6). Given that the dimension of the component matrix **X**_**i**_ is *L* × *K*, further define L* = min(*L*, *K*), K* = max(*L*, *K*), and transform the matrix **X**_**i**_ into a sequence [*z*_1_, *z*_2_, ⋯, *z*_*N*_] of length N by the following diagonal average formula:
    $$\begin{array}{c} z_{k}=\{\begin{array}{c} \frac{1}{k}\sum_{m=1}^{k}x_{m,k-m+1}1\leq k<L^{*}\\ \frac{1}{L^{*}}\sum_{m=1}^{L^{*}}x_{m,k-m+1}L^{*}\leq k\leq K^{*}\\ \frac{1}{N-k+1}\sum_{m=k-K^{*}+1}^{N-K^{*}+1}x_{m,k-m+1}K^{*}<k\leq N \end{array} \end{array}$$ (7)
  (b) Local preferences based on autocorrelation functions
    It is known that HR-related signals in rPPG signals are essentially periodic (or at least quasi-periodic). Therefore, based on global optimization results, the periodicity of HR-related signals is used as a priori information to select the most periodic components of the first *s* signals. Thus the autocorrelation coefficient is adopted as the screening criterion of the periodic metric. The autocorrelation coefficient *P*_*i*_(*k*) of the ith component is defined as follows:
    $$\begin{array}{c} P_{i}\left(k\right)=\frac{1}{{\sigma_{i}}^{2}}\sum_{t=0}^{n-k}\left\{z_{i}\left(t\right)-\mu_{i}\right\}\left\{z\left(t_{i}+k\right)-\mu {}_{i}\right\}\left(i=1,2,\ldots ,s\right) \end{array}$$ (8)
    Where *z*_*i*_(*t*) is obtained from the corresponding component matrix **X**_*i*_ by diagonal averaging, *μ*_*i*_ and ${\sigma_{i}}^{2}$ are the average and variance of *z*_*i*_(*t*), respectively, *z*_*i*_(*t*+ *k*) represents the sequence obtained by shifting the elements in *z*_*i*_(*t*) backward to *k* positions. By definition, when *k* = 0, *P*_*i*_(*k*) takes the maximum of 1. Fig 8 depicts the autocorrelation within 12 s of the HR-related signal and the noise signal obtained by low-rank sparse decomposition. It can be seen that if the signal is periodic or quasi-periodic, some peaks will appear in the *k*th order autocorrelation. And the more periodic the signal, the greater the peak. Therefore, for the first *s* components screened by low-rank sparse matrix decomposition, the autocorrelation peak of the components related to HR is often more significant than that of the components related to intermittent noise.
    Then the maximum autocorrelation coefficient *ρ*_*i*_ of each componentcan be defined as follows:
    $$\begin{array}{c} \rho_{i}=\underset{2\leq p\leq J_{i}}{\text{max}\;P_{i}\left(k_{p}\right)}\left(i=1,2,\ldots ,s\right) \end{array}$$ (9)
    Where *k*_*p*_ is the displacement order of the *p*th peak in *P*_*i*_(*k*) and *J*_*i*_ is the number of all peaks. Each component can obtain the maximum autocorrelation coefficient corresponding to the current component through Eq (9) (as shown in Fig 9). Fig 10 shows the maximum autocorrelation coefficient of the first *s* components obtained by global optimization, and the shadow part represents the components which *ρ*_*i*_ greater than 0.85. Usually, component *ρ*_*i*_ > 0.8 is selected for reconstruction, which can reduce the HR estimation error of the reconstructed signal [37]. In this paper, the threshold is set to 0.85. The corresponding component with the *ρ*_*i*_ higher than the threshold is retained to eliminate the noise signal with relatively weak periodicity.
4. Signal reconstruction
  The components obtained by autocorrelation screening are weighted and superposed according to the proportion of singular values, and the reconstructed signal *x*_*rc*_ can be expressed as:
  $$\begin{array}{c} x_{rc}=\sum_{i\in S}w_{i}z_{i} \end{array}$$ (10)
  Where *w*_*i*_ is the proportion of singular value corresponding to *z*_*i*_ in the total singular value, the denoised rPPG signal can finally be obtained after the above steps. Fig 11 shows the actual performance of the proposed LA-SSA method. As shown in Fig 11, the waveform denoising effects are compared about the G-channel signal after five-point moving average filtering, EEMD denoising results, and LA-SSA restored waveform. The red box represents the interference of irregular noise caused by motion or illumination changes on the waveform, and the green box represents the denoising effect. It can be seen that the denoising results of the filtered G-channel signal or EEMD are still subject to the interference of noise to varying degrees. However, the noise caused by motion or illumination changes is significantly reduced after using LA-SSA method. Fig 12 compares the rPPG obtained by LA-SSA recovery with the reference PPG waveform and gives the spectrum of the two signals. It can be seen that the high correlation between rPPG and PPG signals and the consistency of HR obtained by the two signals. The results show that LA-SSA effectively removed the irregular noise contained in rPPG and improved the accuracy of HR estimation.

**Table 1:** EALM algorithm.

Input:	**X**.
Initialization:	**A**_0_ = **E**_0_ = 0, **Y**_0_ shown in Eq (6), *μ*_0_ > 0, *ρ* > 1.
Process:	Repeat until convergence:
	Repeat until convergence:
	Given **E**, optimize **A**: **A**_*k*+1_ = *D*_1/*μ*_(**X** − **E**_*k*_ + *μ*_*k*_^−1^**Y**_*k*_).
	Given **A**, optimize **E**: **E**_*k*+1_ = *T*_λ/*μ*_(**X** − **A**_*k*+1_ + *μ*_*k*_^−1^**Y**_*k*_).
	Update **Y**_*k*+1_ = **Y**_*k*_ + *μ*_*k*_(**X** − **A**_*k*+1_ − **E**_*k*+1_).
	Update *μ*_*k*+1_ = *ρμ*_*k*_.
	Update *k* = *k* + 1.
Output:	**A**_*k*_, **E**_*k*_.

## 3 Experiments

### 3.1 Database

**UBFC-RPPG database** [38]: This dataset contains 50 videos, which are composed of two parts. The first part (marked as SIMPLE) contains eight videos under ideal conditions, and participants are required to sit still with their eyes closed during recording. The second part (marked REALISTIC) contains 42 real-world videos. Subjects were asked to play a time-sensitive mathematical game to increase pulse frequency and maintain HR diversity while simulating typical human-computer interaction scenarios. The video was captured by the Roche C920 HD Pro camera, which was placed 1 meter away from the subjects. The videos are not compressed in 8-bit RGB format. The frame rate of 30 fps and the spatial resolution of 640 × 480 pixels. Each video takes about 2 minutes, and the Contec Medical CMS50E collects PPG pulse signals at a sampling rate of 60 Hz. In this paper, only the REALISTIC videos are used, each video with 30s window length and 1s step to extract HR.

**PURE database** [51]: This dataset consists of 10 persons (8 males and 2 females) performing different, controlled head motions in front of a camera. The head motions contain steady (S, The subject was sitting still and looks directly into the camera avoiding head motion), talking (T, the subjects were asked to talk while avoiding additional head motion), slow translation (ST, the images recorded by the camera were displayed on screen and shown to the subjects. A moving rectangle of the size of the face was added to the image, and the subjects were asked to keep their face inside), fast translation (FT, has the same setup as slow translation, except twice the speed of the moving target), small rotation (SR, different targets that were placed at 35 cm around the camera. The subjects were told to look at these targets in a predefined sequence. They were asked to move not only there eyes but orient their head), and medium rotation (MR, has the same setup as for small rotation, but with targets placed 70 cm around the camera resulting in average head angle of 35°). The test subjects were placed in front of the camera with an average distance of 1.1 meters, resulting in a total of 60 video sequences. Each video takes 1 minute and is recorded by the ECO274 CVGE camera with a resolution of 640 × 480 pixels and a frame rate of 30 fps. The PPG pulse signal is recorded by the Contec CMS50E pulse oximeter with a sampling rate of 60 Hz.

The images of subjects appearing in the paper have been agreed by the subjects.

### 3.2 Experimental results

In this paper, the following indicators are used to evaluate the performance of the HR measurement method:

- **Mean Absolute Error(MAE)**: The mean value of absolute error between *HR*_*rPPG*_ estimated by recovered rPPG signal and *HR*_*PPG*_ estimated by reference PPG signal, reflecting the actual situation of the error between the measured heart rate and the proper heart rate.
- **Root mean square error(RMSE)**: The quadratic error between *HR*_*rPPG*_ and *HR*_*PPG*_. It is susceptible to outliers, reflecting the stability of the algorithm. The lower the value is, the more stable the algorithm is.
- **Pearson correlation factor(r)**: The correlation between *HR*_*rPPG*_ and *HR*_*PPG*_ was used to evaluate the correlation between predicted heart rate and real heart rate.

In the LA-SSA method, the maximum autocorrelation coefficient *ρ*_*i*_ is introduced in SSA component selection to measure component periodicity. The weak periodic noise in the rPPG signal can be removed by setting the *ρ*_*i*_ threshold to select the periodic or quasi-periodic components related to the HR signal. In order to investigate the influence of the appropriate threshold on the accuracy of HR estimation under the condition of *ρ*_*i*_ > 0.8. The threshold was further set to 0.8, 0.85, and 0.90 for component screening and reconstruction, respectively. As shown in Table 2, it can be seen that when the threshold is set to 0.85, the reconstructed signal has the highest accuracy for HR estimation. Therefore, 0.85 is used as the standard for periodic components screening in this paper.

**Table 2:** Effect of different threshold on HR estimation accuracy.

Threshold	MAE	RMSE	r
0.80	1.63	3.95	0.98
**0.85**	**1.37**	**3.61**	**0.98**
0.90	1.89	4.02	0.97

In order to improve the quality of the LA-SSA input signal, make the noise components in the signal are minimized before LA-SSA decomposition and reconstruction. In this paper, the performance of the recovered rPPG signal with different input signals to estimate HR is compared. The green channel [14], the chromaticity signal obtained through CHROM [15], the signal obtained through FastICA filtering [13], and the signal obtained through EEMD decomposition [18] and screening were used as the input of LA-SSA, respectively. The accuracy of the HR estimation is shown in Table 3. The results show that the signal processed by EEMD filtering shows higher accuracy for HR estimation after LA-SSA decomposition and reconstruction compared with the other three methods.

**Table 3:** Performance comparison of LA-SSA HR estimation using different input signals.

Method	MAE	RMSE	r
G+LA-SSA	4.15	11.09	0.79
CHROM+LA-SSA	5.04	14.65	0.65
FastICA+LA-SSA	5.24	14.73	0.66
**EEMD+LA-SSA**	**1.37**	**3.61**	**0.98**

**Fig 13:**
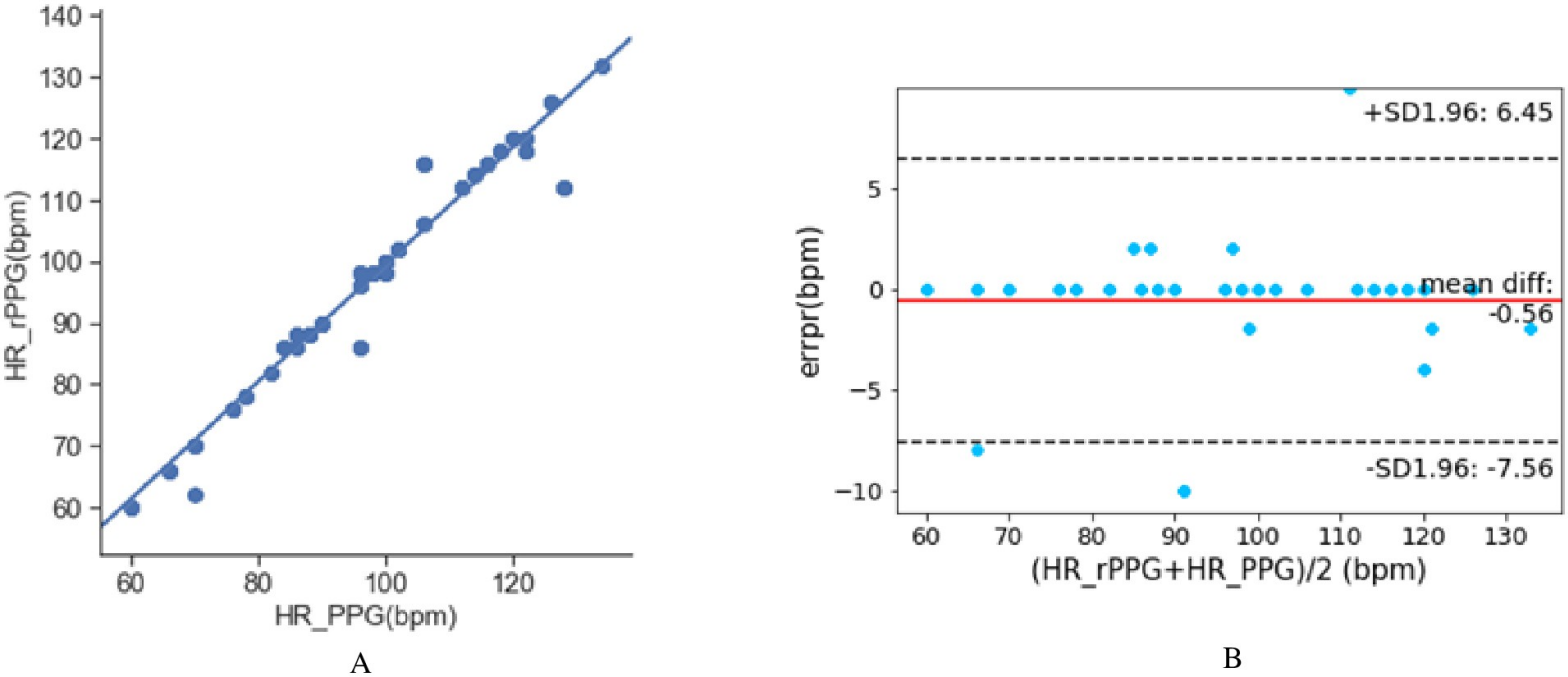
The comparison of HR values obtained by the proposed method and the contact measurement method. A: Linear regression diagram. B: Bland-Altman diagram.

Fig 13 shows the comparison of HR values obtained by the proposed method and the contact measurement method. The linear regression graph is shown in Fig 13A. It can be seen that the data points are concentrated near the linear regression line, and the slope of the linear regression line is close to 1, indicating that the estimated HR is highly correlated with the actual reference HR. Fig 13B is the Bland-Altman consistency analysis diagram. The blue center line represents the relative average error between the heart rate measurement and reference values. Two virtual red lines represent the confidence interval of 95% confidence [*μ* − 1.96*σ*, *μ* + 1.96*σ*], and only the points between the virtual lines are considered to be highly credible. The results show that most of the HR values obtained by the proposed method are in the confidence interval, indicating that the HR measurement values are highly consistent with the reference values.

In the PURE database, there are six different setups corresponding to different levels of head movement noise. Table 4 shows the performance for different noise level of LA-SSA HR estimation. It can be seen that when the setup is steady, the result of HR estimation by the LA-SSA method is best, and the performance may be a little poor when the setup is talking, fast translation and small rotation. However, from the overall results, the proposed method performs well in varying degrees of motion noise in the video.

**Table 4:** Performance for different noise level of LA-SSA HR estimation.

Setup	MAE	RMSE	r
Steady	**1.28**	**6.53**	**0.96**
Talking	3.58	9.40	0.94
Slow Translation	2.45	9.20	0.91
Fast Translation	3.53	9.83	0.90
Small Rotation	3.72	10.65	0.93
Medium Rotation	2.63	9.30	0.93

Finally, Tables 5 and 6 show the performance comparison of the proposed method with other methods on the UBFC-RPPG database and PURE database. In the UBFC-RPPG database, the MAE, RMSE, and Pearson correlation coefficients of the LA-SSA method were 1.37 bpm, 3.61 bpm, and 0.98, respectively, which were better than those of all other algorithms in the table. In the PURE database, the MAE, RMSE, and Pearson correlation coefficients of the LA-SSA method were 2.87 bpm, 7.61 bpm, and 0.96, respectively, which were also better than those of most other algorithms in the table. The results show that the proposed method further removes the irregular noise in rPPG, which effectively improves the accuracy of HR estimation. Several deep learning methods were tested on the two databases. The length of video samples truncated by these deep learning methods is very different from that used in our process.

**Table 5:** Performance of different methods for HR estimation on database UBFC-RPPG.

Method	MAE	RMSE	r
Cpr+fine [40]	2.10	3.43	-
PulseGAN [24]	2.09	4.42	0.97
CK [42]	2.29	3.80	0.98
MAICA [41]	3.34	-	-
POS [42]	2.43	6.60	0.93
ICA [39]	6.02	-	0.79
GREEN [42]	4.47	11.59	0.84
CHROM [24]	3.10	6.84	0.93
cICA [44]	3.14	-	0.91
TSP+CHROM [38]	-	2.38	0.96
Bousefsafet al. [46]	5.45	8.64	-
G-R [43]	9.79	-	0.65
PVM [39]	4.47	-	-
BCG [45]	27.92	37.96	0.25
Tsou et al. [47]	1.29	8.73	-
Lee et al. [48]	5.97	7.42	0.53
PCA [14]	9.65	-	0.67
**EEMD+LA-SSA**	**1.37**	**3.61**	**0.98**

**Table 6:** Performance of different methods for HR estimation on database PURE.

Method	MAE	RMSE	r
ICA [49]	24.1	-	-
CHROM [24]	3.82	-	**0.97**
NMD-HR [49]	8.68	-	-
LICVPR [17]	28.2	-	-0.38
Zhao et al. [50]	3.09	-	-
**EEMD+LA-SSA**	**2.87**	**7.61**	0.96

## 4 Conclusion

In this paper, a new method for SSA components selection and reconstruction is proposed. Based on the contribution of the SSA component to the original signal, the low-rank sparse matrix decomposition is used to select the appropriate reconstructed component in the global component signals. Then combined with the autocorrelation function, the weak periodic noise signal in the reconstructed component is eliminated, and the irregular noise such as face motion and light change in the rPPG signal is effectively removed. Tested on the UBFC-RPPG and PURE dataset, the experimental results verify the best performance of the method. In addition, the autocorrelation function in component filtering is based on the periodic strong and weak screening criteria for noise removal. The motion noise may be identified as the HR correlation component when the human body is in periodic motion, such as fitness. Then the algorithm’s accuracy will be affected to a certain extent. In the subsequent study, we will focus on exploring the recognition and removal method of strong periodic noise. We will do some studies on the approaches based on deep learning.
